# *Phyllanthus emblica* fruit extract alleviates halitosis and reduces the inflammatory response to oral bacteria

**DOI:** 10.1590/1678-7757-2024-0047

**Published:** 2024-06-04

**Authors:** Cheng LU, Liu QING, Lu YINA

**Affiliations:** 1 JAKA Biotechnology Co., LTD Shanghai China JAKA Biotechnology Co., LTD, Tiangong Road No. 818, Jinshan District, Shanghai 201507, China.

**Keywords:** *Phyllanthus emblica* fruit extract, Halitosis-related bacteria, 50% Inhibition concentration, Volatile sulfur compound, Inflammation, Oral care product

## Abstract

**Objective:**

To assess the efficacy of *Phyllanthus emblica* extract in alleviating halitosis and reducing the inflammatory response to halitosis-related bacteria.

**Methodology:**

This investigation, using *Phyllanthus emblica* fruit extract (PE), involved four aspects. First, we evaluated the effect on growth and aggregation of halitosis-related bacteria, including *Fusobacterium nucleatum, Porphyromonas gingivalis*, and *Solobacterium moorei*, using a microdilution assay and scanning electron microscopy. Second, volatile sulfur compound (VSC) levels were measured on individuals with halitosis in randomized short-term (26 participants) and double-blind randomized long-term trials (18 participants in each group) after rinsing with PE for 3, 6, and 12 h, and 28 days. Third, we analyzed pro-inflammatory cytokine expression in TR146 cells using quantitative real-time PCR and enzyme-linked immunosorbent assays. Lastly, we assessed pro-inflammatory cytokine secretion and Toll-like receptor (TLR) 2 mRNA expression via the same experimental methods in a three-dimensional oral mucosal epithelial model (3D OMEM).

**Results:**

PE extract dose-dependently inhibited the growth of *F. nucleatum* (50% inhibition concentration [IC_50_]=0.079%), *P. gingivalis* (IC_50_=0.65%), and *S. moorei* (IC_50_=0.07%) and effectively prevented bacterial aggregation. Furthermore, VSC contents decreased significantly at 3, 6, and 12 h after rinsing with 5% PE compared with those in the control. Long-term use of mouthwash containing 5% PE for 28 days led to a significant decrease in VSC contents. PE attenuated the *F. nucleatum*- or *P. gingivalis*-stimulated mRNA expression and protein release of interleukin (IL)-6 and IL-8 in TR146 cells. It also suppressed IL-8 and prostaglandin E2 secretion and TLR2 mRNA expression in *F. nucleatum*-induced OMEMs.

**Conclusion:**

Our findings support the use of PE in oral care products to alleviate halitosis and it may reduce inflammation.

## Introduction

Halitosis, also known as oral malodor, refers to an unpleasant smell that emanates from the oral cavity. According to surveys, at least 30% of individuals experience chronic oral malodor, with roughly half of them experiencing associated mental health challenges and social embarrassment.^1,2^ Halitosis can be categorized into real halitosis, pseudo-halitosis, and halitophobia.^3^ Real halitosis encompasses two subtypes: physiologic and pathologic halitosis.^4^ Pathologic halitosis can stem from various oral or non-oral factors. Notably, 80–90% of oral malodor originates within the oral cavity.^5^ Moreover, 80–90% of intra-oral halitosis can be attributed to local bacteria in the oral cavity.^6^ This stems from the protein degradation process initiated by anaerobic bacteria in the oral cavity, which utilize nutrition from food debris, exfoliated epithelial cells, and blood. Volatile sulfur compounds (VSC), end products of protein metabolism by oral bacteria, are the main substances closely associated with halitosis and can influence oral health owing to their toxic effects.^7^

The oral cavity is inhabited by VSC-producing, anaerobic gram-negative bacteria, such as *Porphyromonas gingivalis, Fusobacterium nucleatum, Forsythia suspensa*, and *Porphyromonas endodermis. F. nucleatum* and *P. gingivalis* are particularly noteworthy for their copious production of VSC and are considered typical bacteria for halitosis.^8^ Additionally, the gram-positive anaerobic bacterium *Solobacterium moorei,* which deglycosylates glycoproteins via its β-galactosidase activity, provides nutrients for VSC-producing bacteria and is unique to individuals with halitosis.^9^

The homeostasis between the microbiome and the host benefits human health.^10^ The oral mucosal epithelium, which is composed of epithelial cells, protects against physical and chemical damage, microorganisms, and toxins with its structured immune barrier system.^11^ However, an overgrowth of anaerobic and gram-negative bacteria in the oral cavity disrupts this balance, leading to oral malodor and resulting in oral inflammation and periodontal diseases when they infect hard and soft tissues.^12^ *F. nucleatum* and *P. gingivalis* possess virulence factors that selectively target the immune inflammatory response of the host. This mechanism enables their survival in harsh environments and poses significant challenges to the survival of host cells. *F. nucleatum,* one of the most prevalent species in the human gingiva, possesses various virulence factors, including endotoxins (such as lipopolysaccharide [LPS]), adhesins, and serine proteases.^13^ The LPS of *F. nucleatum* can induce the release of pro-inflammatory cytokines, including interleukin (IL)-8, matrix metalloproteinases, and IL-6, which leads to immune responses and further damage to periodontal tissues via nuclear factor-kappa B (NF-κB) activation and nuclear translocation.^14^ *P. gingivalis* also possesses various virulence factors, including fimbriae, proteolytic enzymes, nucleoside diphosphate kinase, capsule, LPS, ceramide, and outer membrane vesicles.^15^ This well-adapted opportunistic colonizing bacterium can invade gingival epithelial cells^16^ and promote the release of pro-inflammation cytokines, including IL-6, IL-8, and tumor necrosis factor-α via Toll-like receptor 4 (TLR4)/TLR2 in human gingival cells.^17^ Additionally, prostaglandin E2 (PGE2) has been detected in inflamed human dental pulp and periapical tissue.^18^

Oral care products, such as toothpaste and mouthwash, offer various options for effectively combatting oral malodor. Chemical antimicrobials, including chlorhexidine, cetylpyridinium chloride, and triclosan, are commonly used in these products; nevertheless, they show potentially harmful effects.^19^ Consequently, focus on exploring and applying new antimicrobials derived from edible botanical sources has been recently increasing. For example, extensive research has been conducted on the antibacterial, halitosis-suppressing, and anti-inflammatory properties of green tea extract and its constituents.^20^ Recent researches have demonstrated the diverse benefits of *Phyllanthus emblica L.* (Indian gooseberry, *Emblica officinalis* Gaertn), including its capacity to inhibit the growth of caries-causing bacteria, prevent and treat oral cancer, and serve as an adjunct in periodontitis therapy.^21,22^ Moreover, short-term chewing of gum containing *Phyllanthus emblica* can stimulate saliva fluidity, counter VSC production, and significantly lessen microbial abundance in saliva.^23^ However, research on the long-term use of *Phyllanthus emblica* in treating halitosis and its effect on associated oral inflammation is scarce. This study investigated the effect of the botanical extract on alleviating halitosis and inhibiting inflammation induced by halitosis-related gram-negative bacteria. This study provides a more comprehensive research base for the application of *Phyllanthus emblica* in oral health care.

## Methodology

### Preparation of PE

The extraction process was performed in our laboratory. Dried *Phyllanthus emblica* fruits (Lincang City, Yunan Province, China) were crushed and subjected to extraction using water at 80–85°C for 2 h. The extract was subjected to adsorption using a chromatographic column composed of D101 and NKA-2 macroporous resins (Sunresi, China). The fraction eluted using 60% glycerin was collected and the solvent evaporated to obtain *Phyllanthus emblica* fruit extract (PE) with a solid content of 1.6%. Total phenolic compounds content is greater than or equal to 1%.

### Bacterial experiments

#### Bacteria culture and growth conditions

*F. nucleatum* (American Type Culture Collection 10953) and *S. moorei* (German Collection of Microorganisms and Cell Cultures GmbH 22971) were grown in a modified peptone yeast extract glucose medium (Shandong Tuopu, China) supplemented with hemin (Sigma, USA, 5 mg/mL) and vitamin K (Sigma, USA, 0.1 μg/mL). *P. gingivalis* (American Type Culture Collection BAA-308) was grown in a tryptic soy broth medium (BD Difco, USA) supplemented with 5 g/L yeast extract (Sigma, USA), hemin (5 mg/mL), and vitamin K (0.1 μg/mL). *F. nucleatum, P. gingivalis*, and *S. moorei* were incubated in an anaerobic bag for 18 h, 24 h, and 48 h, respectively, at 37°C. The growth of bacterial cultures was monitored at 600 nm optical density (OD_600_) using a Spark microplate reader (TECAN, Switzerland).^24^ Thalli were collected by centrifugation, and Gram staining and microscopic observation were used to verify bacterial purity.

#### Minimal inhibitory concentration and 50% inhibition concentration determination

Minimal inhibitory concentration (MIC) and 50% inhibition concentration (IC_50_) of the PE solution were determined via microplate dilution assay.^25^ *F. nucleatum, P. gingivalis*, and *S. moorei* were resuspended separately in a fresh growth medium and added into a 96-well microplate (Corning, USA) at 100 μL/well, with final cell densities of 2×10^6^ CFU/mL, 5×10^7^ CFU/mL, and 5×10^6^ CFU/mL, respectively. The effects of PE on bacterial growth were evaluated by measuring changes in OD_600_ value using the Spark microplate reader after adding varying concentrations of PE (100 μL/well, ranging from 0.003% to 3%) into the 96-well microplate, followed by anaerobic culturing at 37°C for 18 h, 24 h, or 48 h. The experiments were conducted at least in triplicates. The antimicrobial efficacy of PE was quantitatively evaluated based on the MIC by visual turbidity detection and IC_50_ values according to the growth inhibition curves.

#### Observation of bacterial growth and aggregation

*F. nucleatum, P. gingivalis*, and *S. moorei* were resuspended separately in fresh medium, with final bacterial densities of 1×10^7^ CFU/mL, 8×10^7^ CFU/mL, and 6×10^7^ CFU/mL, respectively. Each bacterial suspension was combined with a specific concentration of PE solution and placed in a 6-well microplate (1.5 mL/well) containing a sterile plastic coverslip. After anaerobic culturing (*F. nucleatum* for 24 h, *P. gingivalis* for 120 h, and *S. moorei* for 72 h), the liquid supernatant was discarded. The bacteria-overlain coverslips were gently washed twice with phosphate-buffered saline and fixed in an electron microscope fixative (Servicebio, China) overnight at 4°C. After dehydration and drying, images were captured using a scanning electron microscope (HITACHI, Japan).

## *In vitro* experiments

### Bacterial stimulation preparation

*F. nucleatum* and *P. gingivalis* were grown in anaerobic bags at 37°C for 18 h or 24 h until they reached the exponential growth phase. The density of heat-inactivated *F. nucleatum* and *P. gingivalis* used for the TR146 cell irritation experiment, achieved by heating at 85°C for 1 h,^26^ was adjusted to 1×10^7^ CFU/mL and 3×10^7^ CFU/mL, respectively. Moreover, the density of viable *F. nucleatum* used for the oral mucosal epithelial model (OMEM) irritation experiment was adjusted to 5×10^7^ CFU/mL as an irritant.

### TR146 cell culture and treatment

The oral epithelial cell line TR146 (derived from squamous oral mucosa carcinoma, BioCell Biotech, China) was cultured in T175 TC-treated cell culture flasks (Thermo Fisher, USA) at 37°C, 5% CO_2_, and 95% humidity.^27^ The culture medium comprised high-glucose Dulbecco’s Modified Eagle Medium (Basal Media, China) supplemented with 15% fetal bovine serum (Gibco, USA) and 1% penicillin/streptomycin (Bioind, Israel). Media replacement was performed every 2–3 d. Next, TR146 cells were seeded into 6- or 96-well microplates (5×10^5^ cells/well and 2×10^4^ cells/well in 6- and 96-well microplates, respectively). After overnight incubation, TR146 cells were treated with heat-inactivated nucleated *F. nucleatum* and *P. gingivalis,* with or without the presence of PE, for 24 h at 37°C.

### Three-dimensional oral mucosal epithelial model resuscitation and treatment

Reconstructed three-dimensional OMEM (3D OMEM) purchased from Guangdong BioCell Biotechnology Co, Ltd (China) emulates stratified squamous epithelial structures and using oral epithelial cell line TR146 as seed cells.^28^ Cells are cultivated on a polycarbonate film established on a Transwell chamber at the air-liquid interface in a chemically defined culture medium, finely adjusted to form epithelial tissue devoid of stratum corneum *in vitro*. The resulting 3D OMEM closely mirrors the structure and function of human oral mucosal epithelium. The OMEMs supported by semi solid culture medium were transported under low temperature. Upon receipt, OMEMs shipped on agar were transferred to a 6-well plate with 0.9 mL of pre-added resuscitation liquid. They were then incubated at 37 °C, 5% CO_2_, and 95% saturated humidity for 1 h. Next, the media were replaced with an equal volume of fresh resuscitation liquid and incubation continued at 37°C and 5% CO_2_ for 18 h±2 h. After overnight resuscitation, activated *F. nucleatum* was added to each well of the model surface with or without the PE solution. The plates, now containing fresh culture (0.9 mL/well), were incubated for another 24 h at 37°C.

### Enzyme-linked immunosorbent assay

Following treatment, culture supernatant fluid from TR146 cells and OMEMs was collected. Next, enzyme-linked immunosorbent assay (ELISA) was employed to measure the release of IL-6 and IL-8 from TR146 cells, as well as IL-8 and PGE2 from OMEMs. Human IL-6 and IL-8 ELISA kits (NeoBioscience, China) and PGE2 ELISA kit (Cayman, USA) were used according to the manufacturer’s instructions. The experiments were conducted at least in triplicate.

### RNA preparation and quantitative real-time PCR

The RNA extraction was conducted using the MiniBEST Universal RNA Extraction kit (TaKaRa, Japan). After quantifying RNA using Qubit (Thermo Fisher, USA), reverse transcription was performed using the PrimeScrip RT reagent kit (TaKaRa, Japan). The TB Green Premix (TaKaRa, Japan) and QuantStudio 3 (Thermo Fisher, USA) were employed for quantitative real-time PCR (qPCR). The results were analyzed using instrumental analysis software. The following primer sequences were used: Glyceraldehyde-3-phosphate dehydrogenase F (GAPDH) (TGACAACTTTGGTATCGTGGAAGG), GAPDH R (AGGCAGGGATGATGTTCTGGAGAG), TLR2 F (ATCCTCCAATCAGGCTTCTCT), TLR2 R (GGACAGGTCAAGGCTTTTTACA).

All qPCR experiments were conducted in triplicate, and the relative mRNA expression level of the target gene was determined by comparing it with that of GAPDH.

## Clinical experiments

### Experimental sample

The experimental mouthwash sample comprised 5% PE solution in distilled water, whereas the control mouthwash sample comprised distilled water. Before the clinical trial, the experimental sample underwent a 24-h patch safety test, which was approved by the Cosmetics Evaluation Ethics Committee (JKC-2304-00034) of JAKA (Shanghai, China) and performed by the analysis and testing department of JAKA Biotechnology Co., LTD.

### Participants

Initially, 40 individuals with halitosis, aged between 18 and 70 years, were enrolled. The screening criteria for halitosis was a VSC value˃150 ppb in the oral cavity, assessed using a Halimeter (Interscan Corporation, USA^24^). At the start, 30 participants were selected randomly for the short-term clinical trial. However, only 26 participants (13 females and 13 males) with halitosis were included in the study, since four participants withdrew from the trial for personal reasons during the trial. Additionally, 36 participants (14 females and 22 males) were selected randomly for the long-term clinical trial. The studies were approved by the Cosmetics Evaluation Ethics Committee (JKC-2304-00033) of JAKA. Before testing, informed consent was obtained from each participant.

### Short-term clinical trial

On the day preceding the trial, the participants completed oral hygiene before 10 p.m. and refrained from eating thereafter. On the day of the trial, the participants were instructed to abstain from breath-freshening products and food. The Halimeter was used to measure the initial VSC contents in the oral cavity three times. Timing commenced after participants had completed rinsing with the 20 mL control sample for 30 s, and the VSC contents were reevaluated at 3, 6, and 12 h. After a 1-d interval, the same participants were instructed to follow the same testing requirements and protocol; however, the control sample was replaced with the experimental sample. During the 2-d testing period, the participants maintained the same mild-flavored diet, refrained from using breath freshening products, and were assessed at the same time points as for the control.

### Long-term clinical trial

A total of 36 participants were divided randomly into a control group and an experimental group, with 18 participants in each group. The study was designed for a double-blind randomized controlled trial. On the day before testing day, the participants completed oral hygiene before 10 p.m. and refrained from eating thereafter. On the day of testing, the participants were instructed to abstain from breath-freshening products and food. The Halimeter was used to measure the initial VSC contents in the oral cavity thrice on the day of the first visit. Participants were distributed with the mouthwash samples (control or experimental) and were instructed to rinse with them three times (20 mL for 30 s/time) a day after meals for 28 days. They were instructed to maintain the same oral hygiene habits they had prior to the trial. After 28 days, the participants followed the same testing requirements and protocol on the day before and on the day of testing.

## Statistical analysis

One-way analysis of variance test followed by Dunnett’s multiple comparisons test (α=0.05) were performed for within-group comparisons in *in vitro* experiments. Wilcoxon signed-rank test (α=0.05) and paired two-tailed Student’s *t*-test (α=0.05) were used to compare differences between PE and control at different time points in the short-term clinical trial (n>20), following the Shapiro-Wilk test. Wilcoxon signed-rank test (α=0.05) was used to analyze significant differences before and after rinsing for 28 days in the long-term clinical trial (n<20). All data are expressed as mean±standard deviation of at least three replicates. Statistical significance was defined as P≤0.05 (*), P≤0.01 (**), and P≤0.001 (***).

The change in VSC content was calculated as follows:


ΔVSC content =VSC content after use-VSC content before use (1)

Alleviation was determined using the following formula:


 Alleviation (%)=(△VSC content of PE−△VSC content  of control )/ΔVSC content of control ×10 (2)

## Results

### Bacterial growth and aggregation inhibition

**Figure 1:**
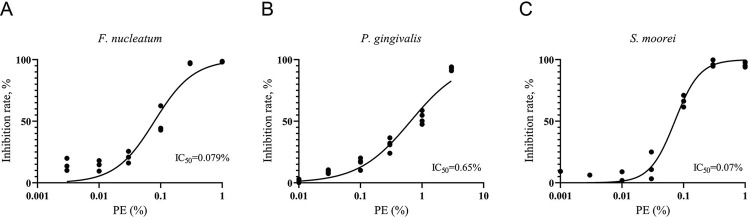
The growth inhibition curves and calculated 50% inhibition concentration values of the *Phyllanthus emblica* fruit extract (PE) solution for (A) *Fusobacterium nucleatum*, (B) *Porphyromonas gingivalis*, and (C) *Solobacterium moorei*. All data presented is comprised of at least three replicates.

The PE produced a dose-dependent growth inhibition of *F. nucleatum, P. gingivalis,* and *S. moorei*. The IC_50_ values of PE against these bacteria were 0.079%, 0.65%, and 0.07%, respectively, whereas the MIC values were 0.3%, 3%, and 0.3%, respectively (Figure 1).

**Figure 2:**
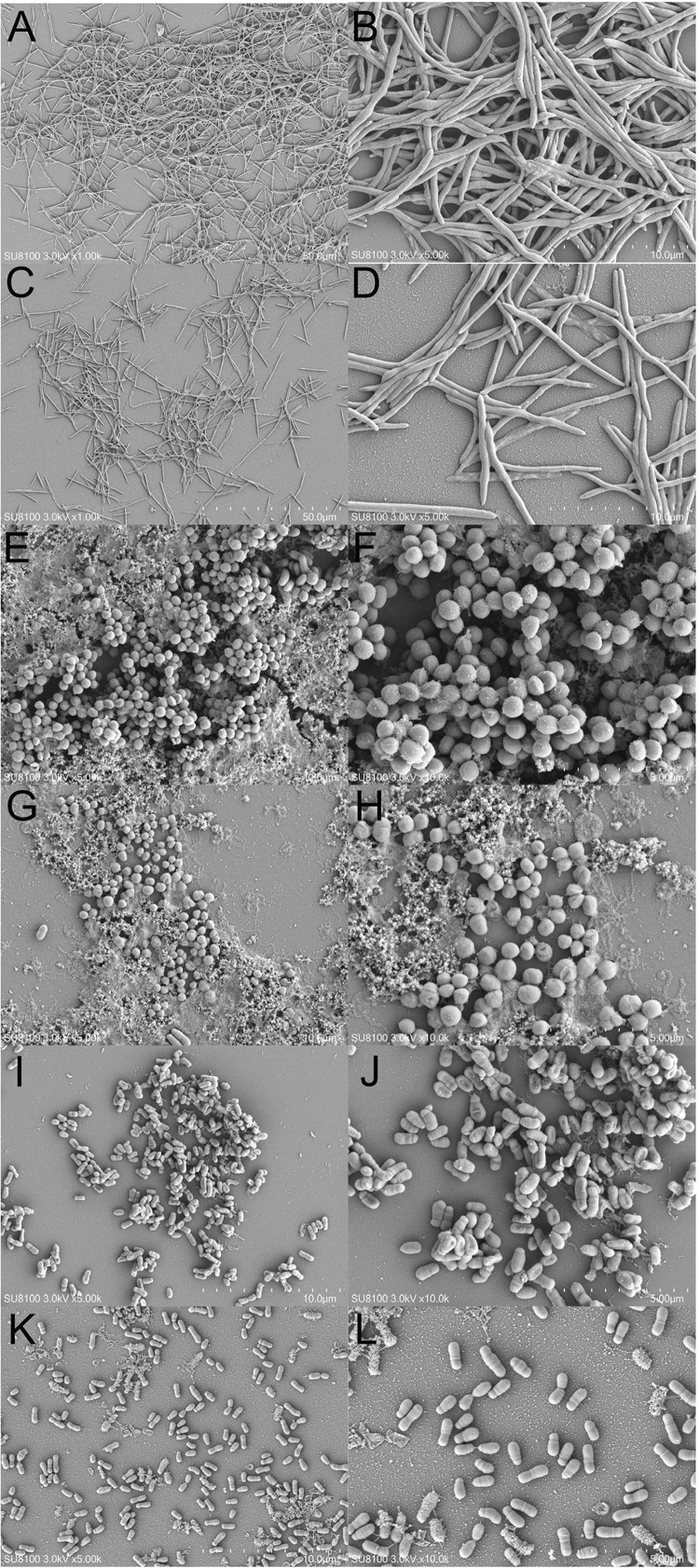
Scanning electron microscopy of bacterial aggregation. (A) 24-h aggregation of *F. nucleatum*; (B) 24-h aggregation of *F. nucleatum*; (C) 24-h aggregation of *F. nucleatum* treated with 0.01% PE; (D) 24-h aggregation of *F. nucleatum* treated with 0.01% PE; (E) 120-h aggregation of *P. gingivalis*; (F) 120-h aggregation of *P. gingivalis*; (G) 120-h aggregation of *P. gingivalis* treated with 0.3% PE; (H) 120-h aggregation of *P. gingivalis* treated with 0.3% PE; (I) 72-h aggregation of *S. moorei*; (J) 72-h aggregation of *S. moorei*; (K) 72-h aggregation of *S. moorei* treated with 0.01% PE; (L) 72-h aggregation of *S. moorei* treated with 0.01% PE

Observations via scanning electron microscopy revealed that the extent of aggregation increased with bacterial incubation time. Notably, 0.01% PE inhibited the accumulation and aggregation of *F. nucleatum* and *S. moorei* during growth. Furthermore, 0.3% PE impeded the aggregation of *P. gingivalis* during its developmental phase (Figure 2).

### Clinical VSC content tests

**Figure 3:**
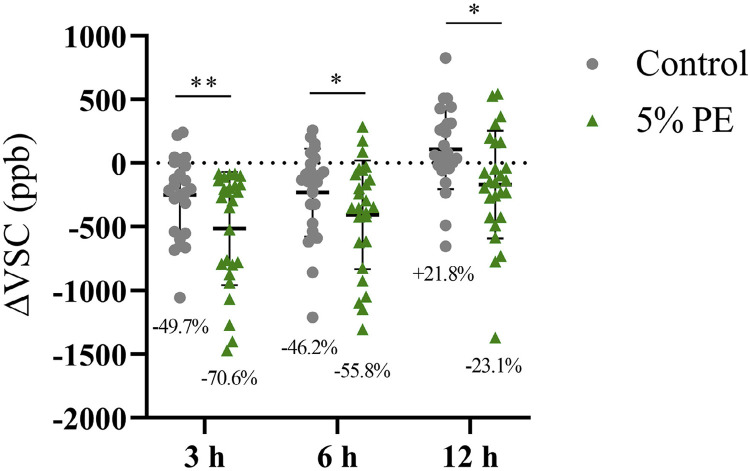
Changes in volatile sulfur compound contents measured using a Halimeter at 3, 6, and 12 h after rinsing. All data are represented by a scatter plot and labeled with the mean±SD of 26 participants. Wilcoxon signed-rank test and paired two-tailed Student’s t-test were used for statistical analysis after the Shapiro-Wilk test. P≤0.05 (*) and P≤0.01 (**) vs control group.

**Figure 4:**
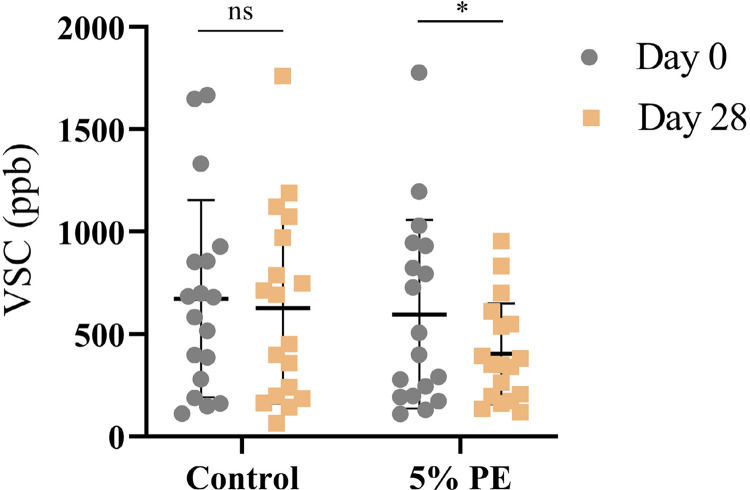
Volatile sulfur compound contents measured using a Halimeter after daily rinsing for 28 d. All data are represented by a scatter plot and labeled with the mean±SD of 18 participants in each group. Wilcoxon signed-rank test was used for statistical analysis. P>0.05 (ns) and P≤0.05 (*) vs day 0.

After using the control mouthwash, VSC contents in the oral cavity decreased by 49.7% and 46.2% at 3 and 6 h time points, respectively (Figure 3). A subsequent increase in VSC contents, rising by 21.8%, was observed as the time post-rinsing increased to 12 h. However, rinsing with 5% PE resulted in a reduction in VSC contents in the oral cavity by 70.6%, 55.8%, and 23.1% at 3, 6, and 12 h post-rinsing, respectively. Results from the experimental PE group exhibited significant differences compared with those from the control group. In the long-term trial, the VSC contents in the oral cavity reduced by 7.1% with no significant difference after 28 days of use in the control group, whereas a significant decrease of 31.6% was observed after rinsing with 5% PE for 28 days (Figure 4). Notably, no adverse events were observed during the testing.

### Anti-inflammatory effects on TR146 cells

**Figure 5:**
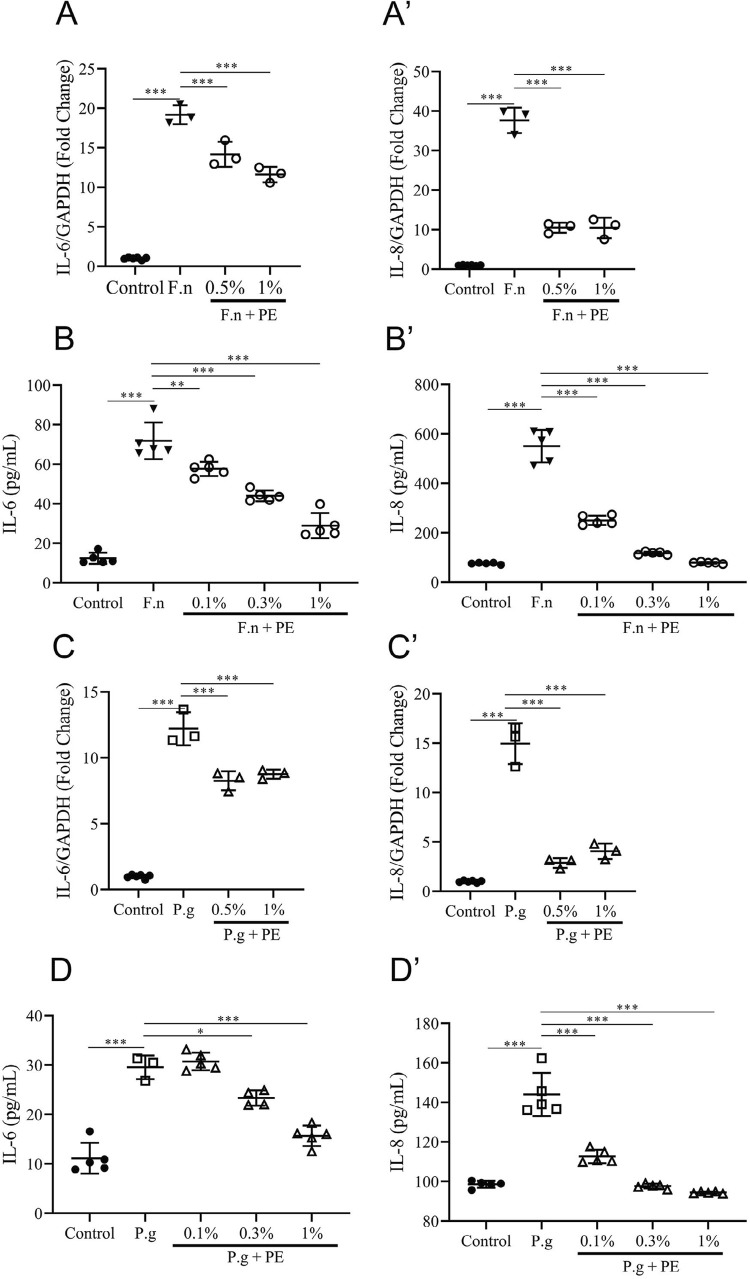
PE inhibited the expression of inflammatory cytokines in TR146 cells after irritation. (A, A’, B, B’) TR146 cells were incubated with inactivated *F. nucleatum* (1×107 CFU/mL) combined with or without PE for 24 h. mRNA expression and protein release were evaluated using quantitative real-time PCR (qPCR) and enzyme-linked immunosorbent assay (ELISA), respectively. (C, C’, D, D’) TR146 cells were cultured with inactivated *P. gingivalis* (3×107 CFU/mL) combined with or without PE for 24 h. mRNA expression and protein release were estimated separately using qPCR and ELISA, respectively. All data are presented as the mean±SD, with scattered points representing each replicate. One-way analysis of variance (ANOVA) was performed for statistical tests. P≤0.05 (*), P≤0.01 (**), and P≤0.001 (***)

To investigate the influence of PE on inflammation induced by halitosis-related bacteria in the oral epithelial cells, we analyzed the expression of pro-inflammatory cytokines stimulated by *F. nucleatum* and *P. gingivalis* for 24 h in TR146 cells using qPCR and an ELISA. The IL-6 and IL-8 mRNA expression and protein release increased significantly after treatment with *F. nucleatum* and *P. gingivalis* compared with those in the untreated controls (Figure 5). However, the addition of PE significantly inhibited the mRNA expression and protein release of IL-6 and IL-8 in TR146 cells with its anti-inflammatory properties.

### Anti-inflammatory effects on OMEMs

**Figure 6:**
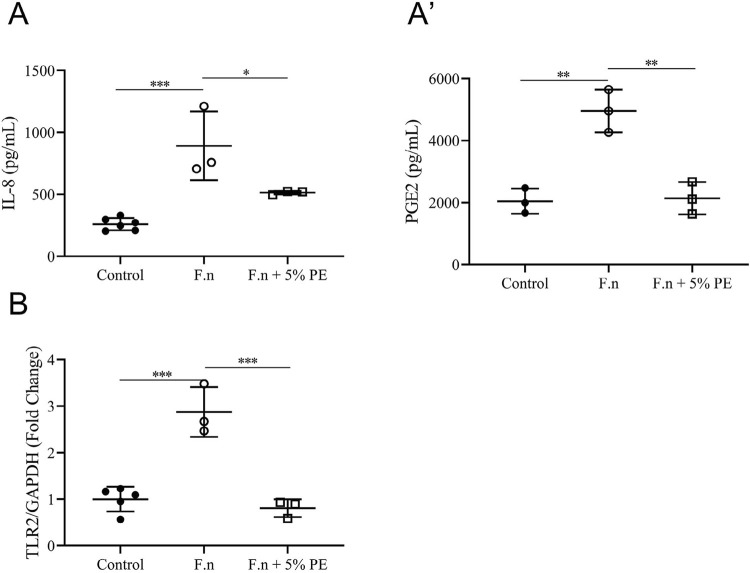
PE inhibited the secretion of pro-inflammatory cytokines and mRNA expression of Toll-like receptor 2 (TLR2) in oral mucosal epithelial models (OMEMs) after irritation. The OMEMs were treated with *F. nucleatum* (5×107 CFU/mL) for 24 h combined with or without PE. (A, A’) Protein expression was measured using ELISA. (B) mRNA level of TLR2 was examined using qPCR. All data are presented as the mean±SD, with scattered points representing each replicate. One-way ANOVA was used for statistical analysis. P≤0.05 (*), P≤0.01 (**), and P≤0.001 (***)

To examine the effect of PE on stimulation induced by halitosis-related bacteria in the oral mucosa, we analyzed the release of pro-inflammatory cytokines stimulated by *F. nucleatum* for 24 h in OMEMs using an ELISA. Additionally, we assessed the mRNA expression of TLR2 in response to inflammatory stimulation caused by *F. nucleatum* using qPCR. The secretion of IL-8 and PGE2 increased significantly after treatment with *F. nucleatum* compared with those in the untreated controls (Figure 6). However, PE suppressed the release of IL-8 and PGE2 in OMEMs with its anti-inflammatory properties. Furthermore, the addition of PE inhibited the *F. nucleatum-*induced up-regulated mRNA expression of TLR2.

## Discussion

*Phyllanthus emblica*, a fruit tree endorsed for global cultivation by the WHO, stands out as a potential edible medicine with extensive applications.^29^ Rich in polyphenols and vitamins, it is extensively used for preventing and treating hypertension, fatty liver, hyperlipidemia, gingivitis, diabetes, and other diseases, owing to its antibacterial, antioxidant, anti-inflammatory, and antimutagenic activities.^30^ Halitosis is mostly attributed to the production of VSCs by anaerobic bacteria in the gingival sulcus and the posterior dorsal region of the tongue, where biofilm formation caused by bacterial aggregation is a pivotal factor in halitosis.^31^ On the one hand, using the gram-negative *F. nucleatum* and *P. gingivalis* and the gram-positive *S. moorei in vitro*, which are contributors to VSC formation, we established the efficacy of PE in alleviating halitosis by antibacterial activity. Our results indicate that PE exhibited dose-dependent growth inhibition activity against *F. nucleatum, P. gingivalis,* and *S. moorei in vitro*. Moreover, PE impeded the accumulation and aggregation of *F. nucleatum, P. gingivalis*, and *S. moorei* during growth. We speculated that PE could alleviate halitosis by reducing the thickness of the microbiota coating of the tongue. On the other hand, we evaluated the influence of PE on VSC contents *in vivo* as a direct indicator of halitosis alleviation. The short-term clinical trial indicated that VSC contents reduced significantly at 3, 6, and 12 h after rinsing with 5% PE compared with those in the control. Long-term use of mouthwash containing 5% PE for 28 days contributed to a significant reduction in VSC contents. The common perception is that oral malodor can be regulated by suppressing the overgrowth of halitosis-associated microorganisms.^32^ Thus, the decrease in VSC contents after long-term use of PE may be due to the effect of PE on halitosis-related bacteria. Furthermore, some researchers have demonstrated that plant polyphenols, when combined with atmospheric oxygen, form orthoquinone, which can react with methyl mercaptan, the main component in VSC, to form non-volatile reaction products.^33^ Phenols, especially caffeic acid, can also reduce the concentration of VSC when combined with oxygen.^34^ Thus, the decrease in VSC contents after short-term use of PE may be attributed to the chemical reaction between polyphenols in PE and VSC.

During the clinical trials, our collaborating dentists and several participants reported that long-term use of PE alleviated oral malodor and mitigated gingivitis. Previous studies showed that with an increase in gram-negative and anaerobic bacteria closely associated with halitosis, endotoxins and lyases can infiltrate the oral mucosa, triggering inflammation and leading to tissue damage.^12,14,33^ Regulating the inflammation caused by halitosis-related bacteria is important for maintaining oral health. Therefore, we established models of oral inflammation related to halitosis using VSC producing bacteria *F. nucleatum* and *P. gingivalis* to verify the effect of PE. We examined the anti-inflammatory effect of PE on *F. nucleatum* or *P. gingivalis*-induced inflammation using TR146 cells. *F. nucleatum* and *P. gingivalis* infection led to an increase in pro-inflammatory IL-6 and IL-8 mRNA expression and protein release, which was subsequently suppressed by PE treatment. Building on this, we selected *F. nucleatum* as the stimulus to establish a pro-inflammatory model on the oral mucosal epithelium, since PE demonstrated similar inhibitory effects on the stimulation induced by *F. nucleatum* and *P. gingivalis. F. nucleatum* infection induced the release of IL-8 and PGE2, which was subsequently suppressed by PE treatment. The TLRs expressed in oral epithelial tissue can recognize *F. nucleatum* and trigger the release of pro-inflammatory cytokines and host defense genes by activating the NF-κB pathway.^35^ *F. nucleatum* has been reported to induce the expression of human beta-defensins in human oral epithelial cells via TLR1, TLR2, and TLR6.^36^ Our study demonstrated that PE treatment effectively inhibited the *F. nucleatum*-induced up-regulated expression of TLR2 in OMEMs. Thus, PE likely influences a series of downstream signaling pathways by regulating TLR2, ultimately exerting anti-inflammatory properties. These findings are consistent with a previous report highlighting the efficacy of PE in oral health owing to the polyphenols with anti-inflammatory effects.^37^

However, our study has several limitations. Although we tried to minimize the differences in testing process between the control and experimental groups in clinical trials, potential biases persist. Additionally, the sample size was relatively small. Thus, further clinical trials with a larger sample size are warranted to assess the impact of long-term use of PE on the overall oral microenvironment. In addition, given the limited data on the molecular mechanism underlying the anti-inflammatory effect of PE, further experimental verification is required to elucidate the specific regulatory mechanism.

## Conclusion

The results of this study demonstrate that PE exhibited antibacterial properties and could alleviate oral malodor. Additionally, it combated inflammation caused by halitosis-related bacteria with its anti-inflammatory activities. These findings suggest its potential as a functional ingredient in oral care products.
